# Case Report: Recognition and management of SMARCA4-deficient renal cell carcinoma

**DOI:** 10.3389/fonc.2025.1614796

**Published:** 2025-11-12

**Authors:** Weibo Wang, Shuiping Yin, Dandan Xu, Supeng Tai, Xi Cheng, Yifan Chang, Yao Fu, Jun Zhou

**Affiliations:** 1 Department of Urology, The First Affiliated Hospital of Anhui Medical University, Hefei, Anhui, China; 2 Institute of Urology, Anhui Medical University, Hefei, Anhui, China; 3 Department of Oncology, The First Affiliated Hospital of Anhui Medical University, Hefei, China; 4 Department of Pathology, The First Affiliated Hospital of Anhui Medical University, Hefei, Anhui, China

**Keywords:** SMARCA4-deficient tumor, renal cell carcinoma, sarcomatoid differentiation, rhabdomyoblastic differentiation, germline mutation

## Abstract

SMARCA4-deficient tumors represent a highly aggressive subtype of malignancy defined by the loss of SMARCA4 expression, and are associated with rapid progression and poor prognosis. We report the first documented case of SMARCA4-deficient renal cell carcinoma (RCC) in an adult (pT3aN1M1, Stage IV), characterized by sarcomatoid and rhabdomyoblastic differentiation, aggressive clinical behavior, and resistance to standard systemic therapies. This case provides a comprehensive analysis of clinical, pathological, imaging, and genetic findings, offering insights to refine diagnostic, therapeutic, and preventive strategies for this rare malignancy.

## Introduction

The SWI/SNF (SWItch/Sucrose non-fermentable) chromatin remodeling complex plays a crucial role in regulating chromatin accessibility and gene expression (1, 2). SMARCA4 encodes BRG1, the core ATPase subunit of this complex that drives chromatin remodeling to regulate gene transcription (3). Recent studies have identified a highly aggressive class of undifferentiated tumors termed SMARCA4-deficient tumors, characterized by SMARCA4 deficiency, and exhibiting rapid proliferation and high metastatic potential, leading to poor clinical outcomes (4). SMARCA4-deficient tumors predominantly arise in the thoracic cavity, sinonasal region, gastrointestinal tract, and female genital tract, but occurrence in the urinary tract, particularly in the kidney, remains exceptionally rare (5–8). Given the significant histological overlap between SMARCA4-deficient tumors and rhabdomyosarcomas, sarcomatoid carcinomas, and other undifferentiated malignancies, accurate diagnosis requires a comprehensive approach incorporating immunohistochemical and genetic evaluation (9–12). Herein, we present the first reported case of SMARCA4-deficient renal cell carcinoma (RCC) in an adult, marked by rapid clinical progression, prominent sarcomatoid and rhabdomyoblastic differentiation, and resistance to standard systemic therapies. Through detailed clinical, pathological, imaging, and genetic analyses, we aim to raise awareness of SMARCA4-deficient RCC, as well as to explore potential diagnostic, therapeutic, and preventive strategies.

## Case presentation

A 71-year-old postmenopausal woman was admitted to our urology service reporting progressive left flank pain radiating to the lower abdominal quadrant persisting for 20 days. Digital palpation disclosed a 4cm fixed mass in the left costo-vertebral angle, demonstrating exquisite tenderness upon deep inspiration.

**Figure 1:**
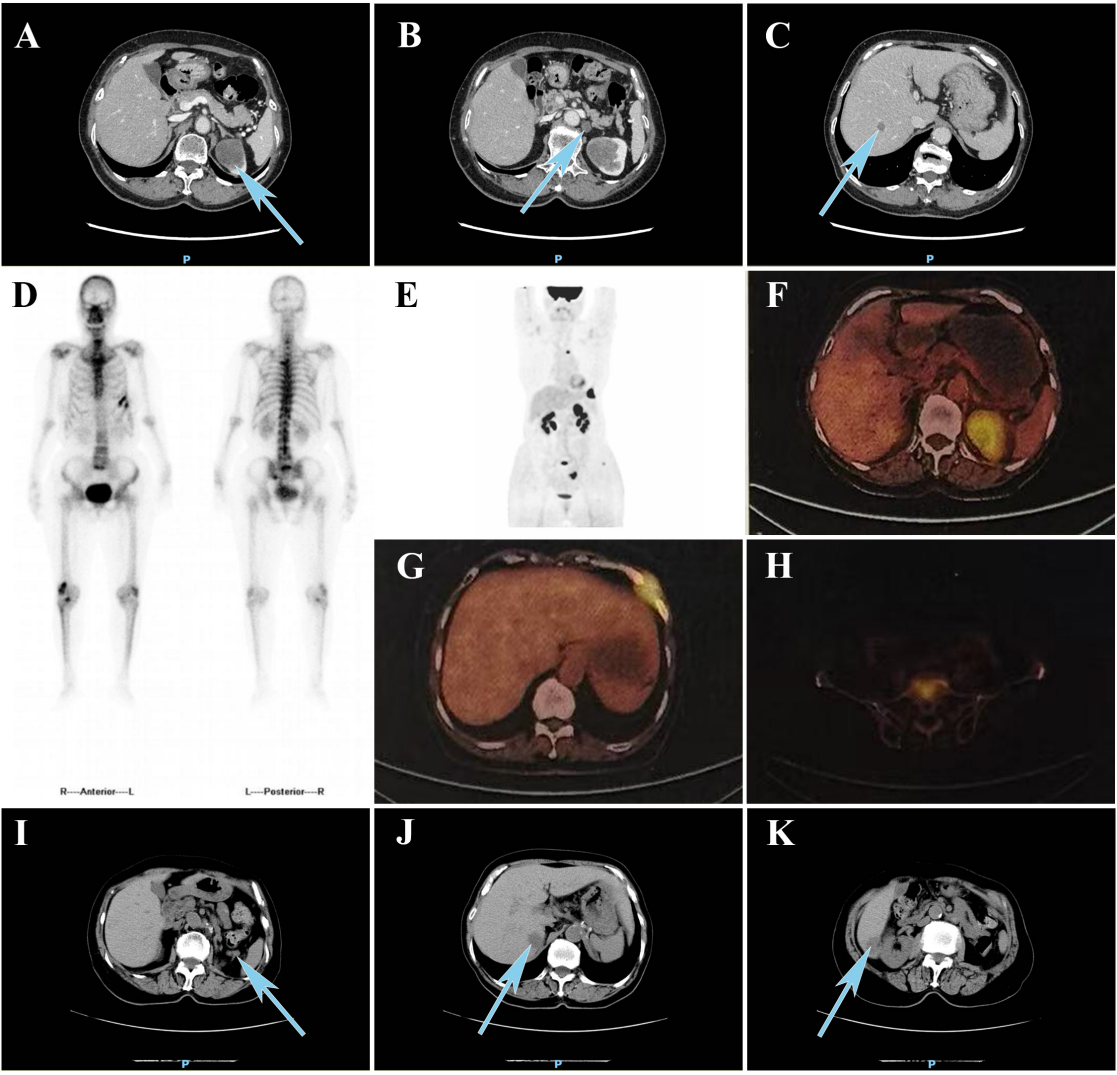
**(A)** Contrast-enhanced CT reveals **(A)** a heterogeneously enhancing mass at the left renal upper pole, **(B)** progressive retroperitoneal lymphadenopathy, and **(C)** hypodense lesions in the liver. **(D)**^99m^Tc-MDP bone scintigraphy demonstrated increased tracer uptake in the left sixth and seventh anterior ribs, sacrum, and left sacroiliac joint. **(E)** MIP reconstruction of ^18^F-FDG PET/CT. ^18^F-FDG PET/CT demonstrated increased fluorodeoxyglucose uptake in **(F)** the upper pole of the left renal upper pole, **(G)** left sixth and seventh ribs, and **(H)** L3 vertebral body. Follow-up CT at 3 months demonstrated newly **(I)** enlarged retroperitoneal lymph nodes and **(J, K)** two hypoattenuating hepatic lesions. CT, computed tomography; 99mTc-MDP, technetium-99m methylene diphosphonate; MIP, maximum intensity projection; ^18^F-FDG, fluorine-18 fluorodeoxyglucose; PET, positron emission tomography.

Laboratory tests showed elevated serum ferritin (311.1 µg/L; reference range: 13–150 µg/L), whereas other tumor biomarkers yielded no significant findings (**Table 1**). Contrast-enhanced abdominal computed tomography (CT) demonstrated a 40 × 36mm heterogeneously enhancing mass at the left renal upper pole, with mild and persistent enhancement accompanied by retroperitoneal lymphadenopathy and subcentimeter hypodense hepatic nodule (**Figures 1A–C**). Technetium-99m methylene diphosphonate (^99m^Tc-MDP) whole-body scintigraphy revealed focally augmented tracer accumulation at left sixth/seventh anterior ribs, sacral alae, and left sacroiliac joint (**Figure 1D**). Subsequent standard Fluorine-18 fluorodeoxyglucose positron emission tomography/computed tomography (^18^F-FDG PET/CT) delineated a hypermetabolic exophytic renal mass (maximum standardized uptake value [SUV_max_] of 7.4) in continuity with the left renal upper pole cortex, accompanied by enlarged retroperitoneal lymph nodes and multiple small hypoattenuating hepatic lesions demonstrating FDG avidity (**Figure 1F**). Osseous metastases were identified in the left sixth and seventh anterior ribs, L3 vertebral body, sacrum, left iliac bone, and left femur, showing metabolically active foci (SUV_max_ 8.5) with CT evidence of variable bone destruction (**Figures 1G, H**).

**Table 1:** Laboratory test results indicate an elevated serum ferritin level.

Item	Result	Reference range	Indication	Unit
AFP	<1.30	0-8.1		ng/mL
CEA	0.77	0-5		ng/mL
GH	0.82	0-10		ng/mL
SF	311.1	13-150	↑	μg/L
CA 125	10.27	0-30.2		U/mL
CA 72-4	0.47	0-12		U/mL
CA 19-9	30.16	0-37		U/mL
CYFRA 21-1	2.11	0-3.3		ng/mL
CA 15-3	3.87	0-32.4		U/mL
β-hCG	0.234	0-5.3		mIU/mL
NSE	15.79	0-17		ng/mL
SCCA	0.38	0-1.5		ng/mL

AFP, alpha-fetoprotein; CEA, carcinoembryonic antigen; GH, growth hormone; SF, serum ferritin; CA 125, carbohydrate antigen 125; CA 72-4, carbohydrate antigen 72-4; CA 19-9, carbohydrate antigen 19-9; CYFRA 21-1, cytokeratin 19 fragment; CA 15-3, carbohydrate antigen 15-3; β-hCG, beta-human chorionic gonadotropin; NSE, neuron-specific enolase; SCCA, squamous cell carcinoma antigen.

**Figure 2:**
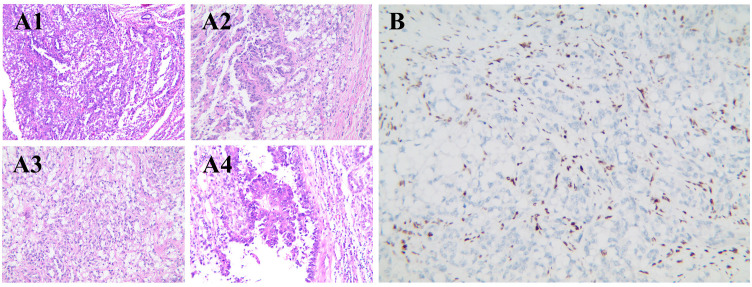
Histologic and immunohistochemical features of SMARCA4-dRCC. **(A1)** Low-power field with tumor cells showing clear to eosinophilic cytoplasm, arranged in solid and focal tubular patterns, with infiltrative borders. **(A2)** High-power field with prominent nucleoli, marked nuclear atypia, and architectural disarray. **(A3)** Sarcomatoid differentiation with spindle-shaped tumor cells in disorganized fascicles. **(A4)** Rhabdomyoblastic differentiation with eosinophilic cytoplasm and eccentric nuclei. **(B)** Immunohistochemistry showing complete loss of SMARCA4/BRG1 expression in tumor cells. RCC, renal cell carcinoma.

The patient underwent da Vinci Xi robotic-assisted radical left nephrectomy with en bloc retroperitoneal lymph node dissection. Postoperative histology revealed a biphasic neoplasm with eccentrically located nuclei and vacuolated cytoplasm, comprising 20% sarcomatoid differentiation and 10% uniform rhabdomyoblastic morphology (**Figure 2A**). Immunohistochemistry showed positive staining for PAX-8, with complete loss of SMARCA4/BRG1 staining (**Figure 2B**). Next-generation sequencing (NGS) identified a heterozygous germline *SMARCA4* (NM_005359.5: c.665C>T, p.Pro222Leu) missense mutation. No clinically actionable biomarkers were detected, as PD-L1 was negative, chemotherapy-related single nucleotide polymorphisms (SNPs) showed no predictive value, and no alterations were found in FDA/NCCN-endorsed targets, indicating resistance to current approved therapeutic options (**Table 2**).

**Table 2:** Genetic mutation analysis results.

Gene	Nucleotide change	Amino acid change	Exon/Intron	Zygosity	Variant allele frequency
Somatic mutation					
NLRP3	c.T2002C	p.F668L	exon3	/	0.5518
TEKT4	c.1212_1213delinsCA	p.A405T	exon6	/	0.0519
KMT2D	c.14888_14889insA	p.P4963fs	exon48	/	0.02
NF2	c.516 + 1G>A	/	exon5	/	0.4897
Germline mutation					
RET	c.G2071A	p.G691S	exon11	Heterozygous	/
ATM	c.A5948G	p.N1983S	exon40	Homozygous	/
CHEK1	c.A1459G	p.I487V	exon12	Homozygous	/
MTHFR	c.C665T	p.A222V	exon5	Heterozygous	/
HNF1A	c.A79C	p.I27L	exon1	Heterozygous	/
POLE	c.C755T	p.A252V	exon8	Heterozygous	/
BRCA2	c.A865C	p.N289H	exon10	Heterozygous	/
BRCA2	c.A2971G	p.N991D	exon11	Homozygous	/
BRCA2	c.T7397C	p.V2466A	exon14	Homozygous	/
MLH3	c.G3488A	p.G1163D	exon5	Heterozygous	/
FANCA	c.A3982G	p.T1328A	exon11	Heterozygous	/
FANCA	c.G4246A	p.G809D	exon26	Homozygous	/
FANCA	c.C1927G	p.P643A	exon22	Homozygous	/
FANCA	c.G1501A	p.G501S	exon16	Homozygous	/
FANCA	c.C1235T	p.A412V	exon14	Heterozygous	/
FANCA	c.A796G	p.T266A	exon9	Homozygous	/
FANCA	c.A4837G	p.S1613G	exon15	Homozygous	/
BRCA1	c.A5483G	p.K1183R	exon10	Homozygous	/
BRCA1	c.A3548G	p.E1038G	exon10	Homozygous	/
BRCA1	c.A3113G	p.P871L	exon10	Homozygous	/
BRCA1	c.C2612T	p.G275D	exon10	Homozygous	/
BRCA1	c.G824A	p.S919P	exon10	Heterozygous	/
BRIP1	c.T2755C	p.P72R	exon19	Homozygous	/
TP53	c.C215G	p.P222L	exon4	Heterozygous	/
***SMARCA4***	***c.C665T***	***p.Q399R***	***exon11***	***Homozygous***	***/***
XRCC1	c.A119G	p.L344V	exon10	Homozygous	/
ERCC2	c.C1030G	p.R119H	exon11	Homozygous	/
POLD1	c.G356A	p.R119H	exon3	Heterozygous	/
DPYD	c.1764delC	p.P588fs	exon14	Heterozygous	/
DPYD	c.A1627G	p.I543V	exon13	Heterozygous	/
DNMT3B	c.C1633T	p.R545C	exon15	Heterozygous	/
ERBB4	c.G2813A	p.R938H	exon23	Heterozygous	/
UGT1A1	c.G211A	p.G71R	exon1	Heterozygous	/
ALK	c.C4587G	p.D1529E	exon29	Homozygous	/
ALK	c.A4472G	p.K1491R	exon29	Homozygous	/
ALK	c.A4381G	p.I1461V	exon29	Homozygous	/
EPCAM	c.T344C	p.M115T	exon3	Homozygous	/
MSH2	c.A505G	p.I169V	exon3	Homozygous	/
ATR	c.G7274A	p.R2425Q	exon43	Homozygous	/
ATR	c.T632C	p.M211T	exon4	Homozygous	/
APC	c.T5465A	p.V1822D	exon16	Homozygous	/
FGFR4	c.G1162A	p.G388R	exon9	Heterozygous	/
SDHA	c.1944-1945del	p.T648fs	exon15	Homozygous	/
PIK3R1	c.G978A	p.M326I	exon8	Homozygous	/
HLA-DPB1	c.A229G	p.K98E	exon2	Heterozygous	/
CFTR	c.C1251A	p.N417K	exon10	Homozygous	/
PRSS1	c.A86T	p.N29I	exon2	Heterozygous	/
PRSS1	c.A508G	p.K170E	exon4	Heterozygous	/
KMT2C	c.G2573T	p.W858L	exon15	Homozygous	/
EGFR	c.G1562A	p.R521K	exon13	Homozygous	/
PMS2	c.A1621G	p.K541E	exon11	Homozygous	/
PMS2	c.C1454A	p.T485K	exon11	Homozygous	/
PMS2	c.C1408T	p.P470S	exon11	Homozygous	/
NBN	c.G553C	p.E185Q	exon5	Heterozygous	/
NOTCH1	c.G3694A	p.V1232M	exon23	Homozygous	/
JAK2	c.G380A	p.G127D	exon5	Heterozygous	/

Bold and italic values indicate the SMARCA4 mutation.

Postoperatively, the patient was treated with a combination of sunitinib and denosumab. At the 3-month follow-up, the patient was clinically stable with no significant limitations in daily activities. However, CT demonstrated progressive retroperitoneal lymphadenopathy and two new hepatic metastases.

## Discussion

The SWI/SNF chromatin remodeling complex governs chromatin structure accessibility and transcriptionally regulates cancer-relevant gene networks by hydrolyzing ATP to mobilize nucleosomes (13). In mammals, the SWI/SNF complex comprises three non-overlapping subtypes, the canonical BAF (cBAF), polybromo-associated BAF (pBAF), and non-canonical BAF (ncBAF) (14). Each subtype contains mutually exclusive ATPase catalytic subunits, either SMARCA4 or SMARCA2, generating mechanical force via ATP hydrolysis for nucleosome mobilization, eviction, or histone exchange (15). This process facilitates the conversion of tightly packed heterochromatin into an accessible, open state, thereby promoting gene transcription. Dysfunctional SMARCA4 impairs SWI/SNF complex assembly, inducing genome-wide enhancer silencing and activating pro-tumorigenic pathways that drive metastasis and therapy resistance (16).

Previous studies have established that SMARCA2 deficiency is a pathognomonic genomic driver in high-grade clear cell renal cell carcinoma (ccRCC), mechanistically linked to epigenetic reprogramming that fuels sarcomatoid differentiation (17). Contrastingly, SMARCA4-deficient tumors predominantly arise in the sinonasal region, thorax, digestive tract, and female genital tract, with occurrences in the urinary tract being exceedingly rare (5–8). Our case represents the first reported instance of SMARCA4-deficient RCC in an adult, pathologically staged as pT3aN1M1 and classified as WHO/ISUP grade 4.

SMARCA4-deficient tumors are typically associated with extremely poor survival outcomes. Carcinoma cells exhibiting hyperactivation of epithelial-mesenchymal transition (EMT), characterized by enhanced migratory and invasive capabilities, which promote the early onset of metastatic spread (18, 19). Clinically, the progression of SMARCA4-deficient tumors occurs at a significantly faster rate compared to other tumor types. The median survival for SMARCA4-deficient gastric carcinoma is less than one year from diagnosis, whereas the median survival for SMARCA4-deficient thoracic sarcomas is only six months from diagnosis (20, 21).

Patients with RCC rarely present with the classic triad of symptoms, including hematuria, flank pain, and abdominal mass in the early stages. More commonly, renal masses are incidentally discovered during abdominal imaging studies conducted for unrelated indications (22). Among imaging modalities, ultrasound is primarily used for tumor screening and adjunctive diagnosis. Contrast-enhanced CT remains the cornerstone for RCC evaluation and multiparametric magnetic resonance imaging (MRI) excels in characterizing cystic/necrotic components and venous invasion, critical for surgical planning (23). Additionally, SMARCA4-deficient tumors and their metastatic lesions typically show a strong affinity for ^18^F-FDG enabling PET/CT to detect occult metastases (24). Although sufficient case data to characterize the specific imaging features of SMARCA4-deficient undifferentiated RCC are currently lacking, their high metastatic potential and metabolic activity suggest that the integration of CT/MRI with PET/CT should be incorporated into diagnostic workflows and clinical staging.

Histologically, SMARCA4-deficient tumors present as infiltrative, diffuse sheets of undifferentiated cells, frequently accompanied by variable rhabdomyoblastic tumor cell components (9). Due to the high degree of dedifferentiation observed in SMARCA4-deficient undifferentiated RCC, these tumors are morphologically similar to SMARCB1-deficient malignant rhabdoid tumor (MRT), rhabdomyoblastic RCC, and other small round cell undifferentiated tumors, which can lead to misdiagnosis (10–12). Therefore, immunohistochemistry is essential, with complete loss of SMARCA4 expression serving as a reliable marker of SMARCA4-deficient tumors.

It is worth noting that we identified a heterozygous germline mutation in SMARCA4 in this patient. Unlike sporadic tumors driven by somatic mutations, this germline alteration follows an autosomal dominant inheritance pattern with incomplete penetrance, indicating a potential hereditary predisposition to cancer (25, 26). This mechanism closely parallels that observed in small cell carcinoma of the ovary, hypercalcemic type (SCCOHT), wherein more than 40% of cases are driven by germline SMARCA4 mutations (27, 28). Thus, we propose that tumorigenesis in SMARCA4-deficient RCC similarly adheres to Knudson’s classic two-hit model: an initial germline loss-of-function mutation serves as the first hit, followed by somatic inactivation of the remaining wild-type allele, leading to complete loss of BRG1 function (29). Given these implications, germline SMARCA4 testing and cascade screening of at-risk relatives are essential to enable tailored surveillance strategies, including periodic imaging and biochemical assessments for early tumor detection and improved clinical outcomes.

SMARCA4-deficient tumors progress aggressively, with over 60% of patients presenting at stage IV by the time of diagnosis (30). For SMARCA4-deficient RCC amenable to surgery, cytoreductive nephrectomy combined with therapeutic options may improve outcomes. Conventional platinum-based chemotherapy shows limited efficacy in these SWI/SNF-altered malignancies due to intrinsic resistance mechanisms (31). Although our case demonstrated no significant evidence of benefit from immunotherapy, studies have shown that more than 65% of such tumors exhibit high PD-L1 expression in both tumor cells and immune cells within the tumor microenvironment (32, 33). Additionally, targeted therapies involving Enhancer of Zeste Homolog 2 (EZH2) inhibitors and histone deacetylase (HDAC) inhibitors have shown promising therapeutic potential in SMARCA4-deficient undifferentiated thoracic tumors (34). Furthermore, Fang et al. recently reported that SMARCA4-deficient cells exhibit upregulation of the oxidative phosphorylation (OXPHOS) pathway, suggesting that OXPHOS inhibitors may serve as potential therapeutic agents for SMARCA4-deficient tumors (35).

During this study, we collected a comprehensive set of clinical, imaging, pathological and genetic data on SMARCA4-deficient RCC, providing insights into potential diagnostic and therapeutic strategies. This case represents the first reported instance of SMARCA4-deficient undifferentiated RCC in an adult, offering valuable contributions to the refinement of future diagnostic, therapeutic, and preventive protocols.

## Data Availability

The original contributions presented in the study are included in the article/supplementary material. Further inquiries can be directed to the corresponding authors.

## Glossary

SWI/SNF: SWItch/Sucrose non-fermentable
CT: computed tomography
^99m^Tc-MDP: technetium-99m methylene diphosphonate
^18^F-FDG PET/CT: fluorine-18 fluorodeoxyglucose positron emission tomography/computed tomography
SUV: standardized uptake value
FH: fumarate hydratase
NGS: next-generation sequencing
SNPs: single nucleotide polymorphisms
BAF: brahma-associated factor
ccRCC: clear cell renal cell carcinoma
WHO: world health organization
EMT: epithelial-mesenchymal transition
MRI: magnetic resonance imaging
MRT: malignant rhabdoid tumor
SCCOHT: small cell carcinoma of the ovary, hypercalcemic type
EZH2: enhancer of zeste homolog 2
HDAC: histone deacetylase
OXPHOS: oxidative phosphorylation
